# 用于复杂生物样品体系分离与识别的分子印迹技术最新进展

**DOI:** 10.3724/SP.J.1123.2024.01011

**Published:** 2024-06-08

**Authors:** Baoxuan XIE, Yang LYU, Zhen LIU

**Affiliations:** 南京大学化学化工学院, 生命分析化学国家重点实验室, 江苏 南京 210023; State Key Laboratory of Analytical Chemistry for Life Science, School of Chemistry and Chemical Engineering, Nanjing University, Nanjing 210023, China

**Keywords:** 分子印迹技术, 分子印迹聚合物, 复杂生物样品, 基质效应, 固相萃取, 分离, 综述, molecular imprinting technology (MIT), molecularly imprinted polymer (MIP), complex biological samples, matrix effect, solid-phase extraction (SPE), separation, review

## Abstract

由生物样品中复杂组分所导致的基质效应会严重影响分离分析技术的准确性、灵敏度与可靠性。免疫亲和技术作为降低或消除基质效应的方法已被广泛应用于诊断分析和蛋白纯化等领域,但该技术仍存在明显的缺点,如成本高昂、制备流程繁琐、保存条件苛刻以及配体浸出等问题。目前,如何通过有效降低或消除复杂生物样品中的基质效应来实现痕量目标分析物的分离及识别仍是一个具有挑战性的问题。分子印迹技术(molecular imprinting technology, MIT)一直被广泛应用于固相萃取与色谱分离等领域,随着MIT的发展,各种新型印迹策略被提出;其中,分子印迹聚合物(molecularly imprinted polymer, MIP)作为一种能够模拟抗原-抗体间相互作用的高分子聚合物,可以从各种复杂生物样品中提取出目标分析物,从而有效消除基质效应的影响。MIP不仅拥有高特异性与高亲和力的优点,而且与抗体和适配体等生物大分子相比,MIP还具有稳定性高、成本低廉以及制备简便等优势。近年来一些基于MIT的传统分离技术得到了深入发展,其中包括色谱固定相以及固相萃取吸附剂等。此外,结合了MIT与高灵敏检测技术的分析方法在疾病诊断和生物成像等领域也受到了广泛关注。本文着重介绍了近年来发展的新型印迹策略,并介绍了基于MIP的分离分析方法在各领域中的应用以及现阶段存在的不足,最后对MIT的未来发展方向做出了展望。

## Abstract (en)

Given continuous improvements in industrial production and living standards, the analysis and detection of complex biological sample systems has become increasingly important. Common complex biological samples include blood, serum, saliva, and urine. At present, the main methods used to separate and recognize target analytes in complex biological systems are electrophoresis, spectroscopy, and chromatography. However, because biological samples consist of complex components, they suffer from the matrix effect, which seriously affects the accuracy, sensitivity, and reliability of the selected separation analysis technique. In addition to the matrix effect, the detection of trace components is challenging because the content of the analyte in the sample is usually very low. Moreover, reasonable strategies for sample enrichment and signal amplification for easy analysis are lacking. In response to the various issues described above, researchers have focused their attention on immuno-affinity technology with the aim of achieving efficient sample separation based on the specific recognition effect between antigens and antibodies. Following a long period of development, this technology is now widely used in fields such as disease diagnosis, bioimaging, food testing, and recombinant protein purification. Common immuno-affinity technologies include solid-phase extraction (SPE) magnetic beads, affinity chromatography columns, and enzyme linked immunosorbent assay (ELISA) kits. Immuno-affinity techniques can successfully reduce or eliminate the matrix effect; however, their applications are limited by a number of disadvantages, such as high costs, tedious fabrication procedures, harsh operating conditions, and ligand leakage. Thus, developing an effective and reliable method that can address the matrix effect remains a challenging endeavor. Similar to the interactions between antigens and antibodies as well as enzymes and substrates, biomimetic molecularly imprinted polymers (MIPs) exhibit high specificity and affinity. Furthermore, compared with many other biomacromolecules such as antigens and aptamers, MIPs demonstrate higher stability, lower cost, and easier fabrication strategies, all of which are advantageous to their application. Therefore, molecular imprinting technology (MIT) is frequently used in SPE, chromatographic separation, and many other fields. With the development of MIT, researchers have engineered different types of imprinting strategies that can specifically extract the target analyte in complex biological samples while simultaneously avoiding the matrix effect. Some traditional separation technologies based on MIP technology have also been studied in depth; the most common of these technologies include stationary phases used for chromatography and adsorbents for SPE. Analytical methods that combine MIT with highly sensitive detection technologies have received wide interest in fields such as disease diagnosis and bioimaging. In this review, we highlight the new MIP strategies developed in recent years, and describe the applications of MIT-based separation analysis methods in fields including chromatographic separation, SPE, diagnosis, bioimaging, and proteomics. The drawbacks of these techniques as well as their future development prospects are also discussed.

随着医学、药学与生物学等领域的快速发展,对复杂生物样品分离分析技术准确性及灵敏度的要求不断提高。在过去的几十年,包括电泳、光谱、色谱、质谱和电化学等在内的许多方法被开发出来,以用于复杂生物样品的分离分析^[1,2]^,然而如何降低或消除血液、尿液及唾液等生物样本的基质效应仍是一个具有挑战性的问题^[3]^。基于抗原-抗体特异性结合的免疫亲和技术是减少或消除基质效应的一种有效方法^[4,5]^,抗原-抗体相互作用的高特异性和高亲和力有利于从各种复杂生物样品中特异性地提取目标分析物。近年来,随着抗体发现技术(如噬菌体展示抗体库、转基因动物以及人源单B细胞等)的不断扩展,理论上可以在各种生物体中实现针对任何靶标抗体的筛选^[6]^。因此,基于免疫亲和技术的固相萃取磁珠、亲和色谱柱以及酶联免疫吸附法(enzyme linked immunosorbent assay, ELISA)试剂盒等被广泛开发出来,并应用于生物医学领域;但免疫亲和技术仍具有一些明显的缺点,包括成本昂贵、制备繁琐、稳定性差以及配体浸出等问题^[7,8]^。因此,针对复杂生物样品基质效应的消除问题,发展一种高选择性的目标分析物分离富集技术仍然十分必要。

分子印迹技术(molecular imprinting technology, MIT)是一门通过模拟抗原-抗体间相互作用来专一性识别目标分析物的技术,其具有高特异性与高亲和力等优点,可有效消除复杂生物样品中的基质效应,从而实现痕量目标分析物的分离与富集^[9,10]^。分子印迹聚合物(molecularly imprinted polymer, MIP)是基于MIT所开发出来的一种化学合成高分子材料,与抗体和适配体等生物大分子相比,MIP具有制备简单、成本低廉以及稳定性好等优势^[11,12]^。如图1所示,MIP的印迹过程一般包括3个部分^[13]^:(1)功能单体与模板分子通过相互作用力结合,形成模板-单体复合物;(2)通过交联剂使功能单体聚合固定在模板周围;(3)将模板分子从聚合物中去除,从而得到对模板分子具有特定识别作用的三维空腔。MIP的模板可以是有机分子、生物大分子甚至是微生物^[14]^,这意味着可以按照实验需求来定制能够特异性识别各种目标分析物的MIP。此外,将MIP与传感器、光谱和质谱等分析方法相结合,可以实现目标物的快速提取分离与高灵敏检测^[15]^。近年来,基于MIP的分离分析方法发展迅速,越来越多的MIP被应用于样品分离、样品制备以及生化分析等领域^[16⇓-18]^。

**图 1:**
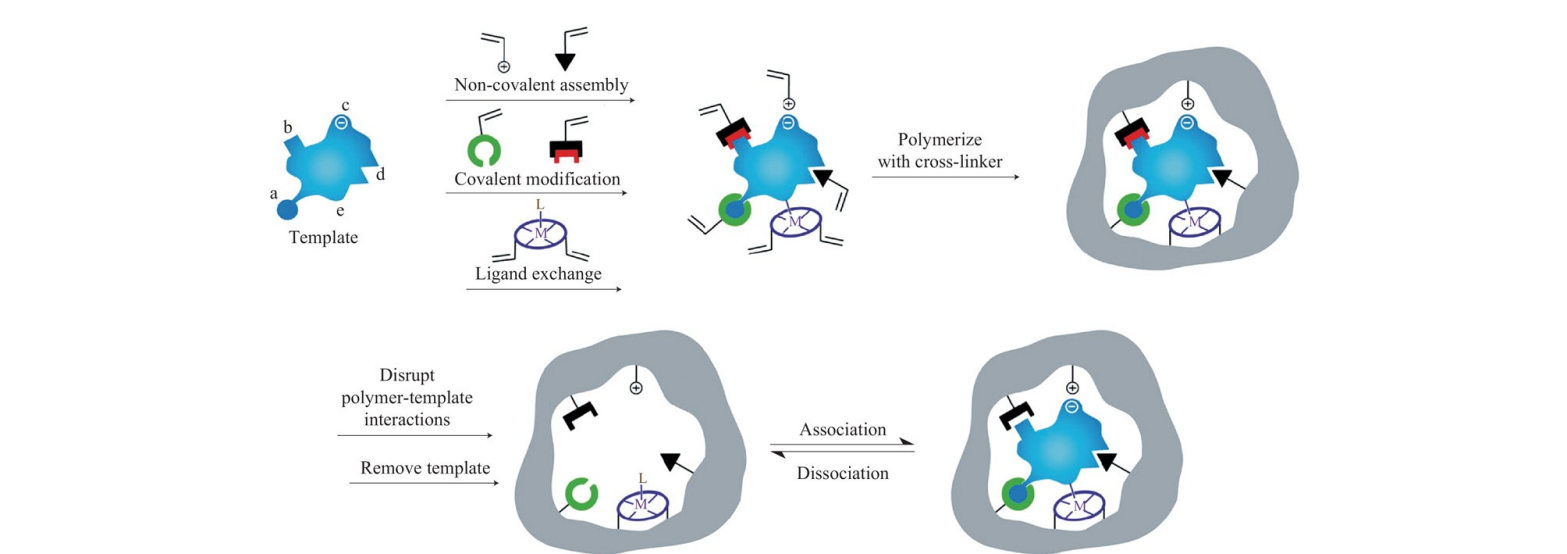
MIT的原理示意图^[13]^

目前,已有不少综述介绍过MIT在生物样品分离分析中的应用,如2020年Silva等^[19]^介绍了MIP作为固相萃取材料在疾病生物标志物分析中的应用,并对MIP在临床样品制备中的挑战与发展趋势进行了总结和展望;2023年Wang等^[20]^系统综述了各类分子印迹比色传感器的制备方法及其在环境分析和食品安全监控等领域中的应用,并总结了各类分子印迹比色传感器在实际应用中的局限性与发展方向。然而,目前大部分综述是针对分离分析中某个特定领域的总结^[17,19,21]^,或是围绕某一类与MIT相结合的技术展开介绍^[20,22,23]^。因此,亟需一篇更为全面的应用概述,帮助读者更加完整地了解MIT在各个研究领域中的应用。本综述分为3个部分,第一部分重点介绍了基于MIP的印迹策略,特别是近年来发展的新型印迹策略;第二部分概述了MIP在各个领域中的应用,主要包括色谱分离、固相萃取(solid-phase extraction, SPE)、疾病诊断、生物成像以及蛋白质组学等领域;最后,本文对MIT在复杂生物样品分离分析应用中的未来发展方向进行了总结与展望。

## 1 分子印迹策略

### 1.1 本体印迹

本体印迹是早期用于制备MIP的一种常规方法,即首先将模板分子、功能单体与交联剂混合均匀,随后加入引发剂进行聚合反应,在洗脱模板后对聚合物进行机器粉碎,通过研磨和筛分得到尺寸合适的小颗粒聚合物^[24,25]^。对于蛋白质、外泌体和微生物等具有复杂结构的模板分子,本体印迹策略存在着很多缺点^[18,25]^。本体印迹所使用的“一锅法”会不可避免地使相当一部分模板包埋于聚合物基质内部,导致模板的去除或目标物的重新结合受到阻碍。此外,在粉碎研磨的过程中,MIP上的印迹位点可能会受到损坏,导致结合性能下降。以上这些局限性严重阻碍了MIP在分离分析应用中的发展,因此,多年来科研工作者们一直致力于发展新型的印迹策略。

### 1.2 表面印迹

为解决上述本体印迹策略的局限性,表面印迹策略被开发出来。与本体印迹不同,表面印迹能够在基质表面形成识别位点,可以有效去除模板分子与结合目标物,并具有更高的模板利用率和更快的响应速度。许多纳米材料都可以作为基质用于表面印迹聚合物的制备^[24]^,如SiO_2_纳米颗粒^[26,27]^、磁性纳米颗粒(magnetic nanoparticles, MNPs)^[28]^以及量子点(quantum dots, QDs)^[29]^等。一方面,纳米材料的引入可以为MIP提供较高的比表面积,从而提高其与目标物的结合能力;另一方面,纳米材料的引入能够赋予MIP更多的特殊性能,如磁响应和荧光发射等。

因此,表面印迹已经替代本体印迹成为一种主流的印迹策略。Saylan等^[30]^在乙烯功能化的SiO_2_纳米颗粒表面以免疫球蛋白G(immunoglobulin G, IgG)为模板、*N*-甲基丙烯酰-L-天冬氨酸(*N*-methacryloyl aspartic acid, MAAsp)为功能单体,通过聚合反应形成IgG印迹薄层,所制备出的印迹薄层对IgG具有较高的选择性和较大的结合容量。Fu等^[31]^利用SiO_2_纳米颗粒为核心,在其表面使用3-氨丙基三甲氧基硅烷和马来酸酐进行改性,从而引入可聚合的双键与羧基,并通过静电相互作用固定溶菌酶(lysozyme, Lys)模板,合成了具有良好结合能力的Lys核壳MIP。然而,早期大部分的表面印迹方法都无法在基质上可控、定向地固定模板分子,这极大阻碍了MIP识别性能的提升。为此,本课题组^[32]^发展出了一种硼亲和可控定向表面印迹方法(图2),该方法依赖硼亲和作用将模板固定在硼酸功能化的基底上,在加入功能单体后通过自聚合反应在基底上形成厚度适当的印迹层。该方法通过简单的模板固定、定向印迹和模板洗脱3个步骤获得了具有良好识别性能的MIP,且所制备的MIP对糖蛋白、聚糖和单糖的解离平衡常数(*K*_d_)分别能达到10^-8^~10^-9^、10^-5^以及10^-4^ mol/L水平。此外,在定向印迹过程中可以通过控制印迹时间来调整印迹层的厚度。

**图 2:**
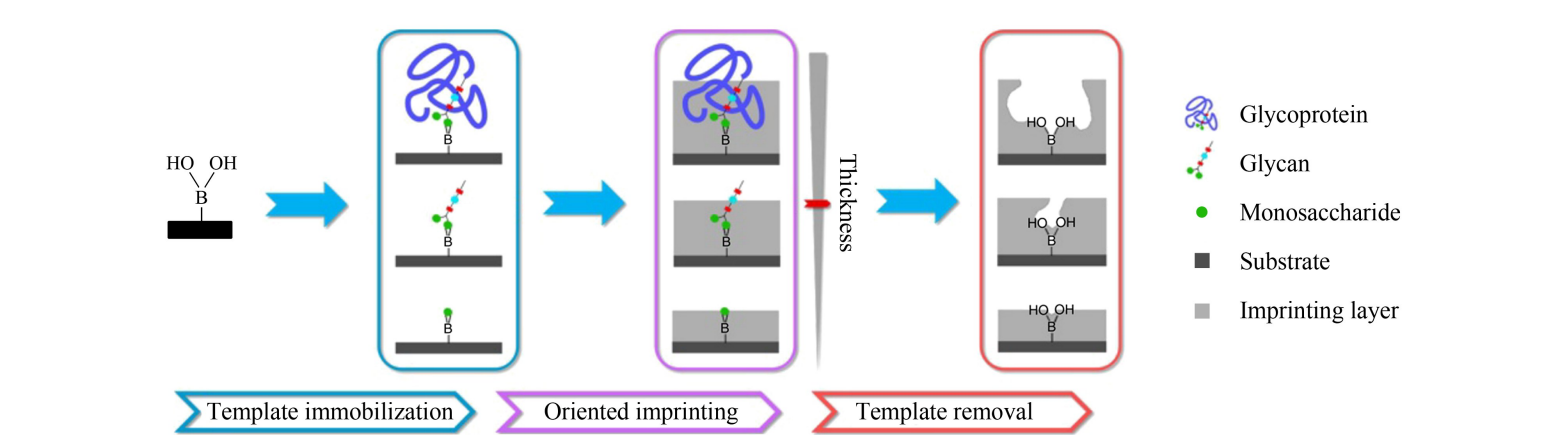
硼亲和可控定向表面印迹技术的示意图^[32]^

然而,上述策略仅适用于糖蛋白、糖肽、聚糖或单糖等各类含顺式二羟基的模板分子,而不能直接应用于不含顺式二羟基的模板分子。为了克服这一缺点,本课题组^[33]^在该策略的基础上提出了一种改进方法,即硼亲和锚定表位可控定向表面印迹技术(图3)。该方法选取蛋白质的C端或N端九肽作为表位,并在表位末端修饰果糖分子,随后利用硼亲和相互作用将糖化表位固定在硼酸功能化的基质上,并通过多种硅烷化试剂进行缩聚反应,以在基质表面形成厚度适当的SiO_2_印迹层。该方法中的硼酸仅用于固定模板,而不参与目标蛋白质的重新结合,因此参与印迹的功能单体除了正硅酸四乙酯(tetraethyl orthosilicate, TEOS)外,还有各种能够与模板形成相互作用的硅烷化试剂,包括氨丙基三乙氧基硅烷(3-aminopropyl-triethoxysilane, APTES)、3-脲丙基三乙氧基硅烷(*N*-(triethoxysilylpropyl)urea, UPTES)以及异丁基三乙氧基硅烷(isobutyl-triethoxysilane, IBTES),这些功能单体能够与不同的氨基酸形成静电相互作用、氢键和疏水相互作用等作用力,这些作用力对于形成高亲和力的MIP至关重要。

**图 3:**
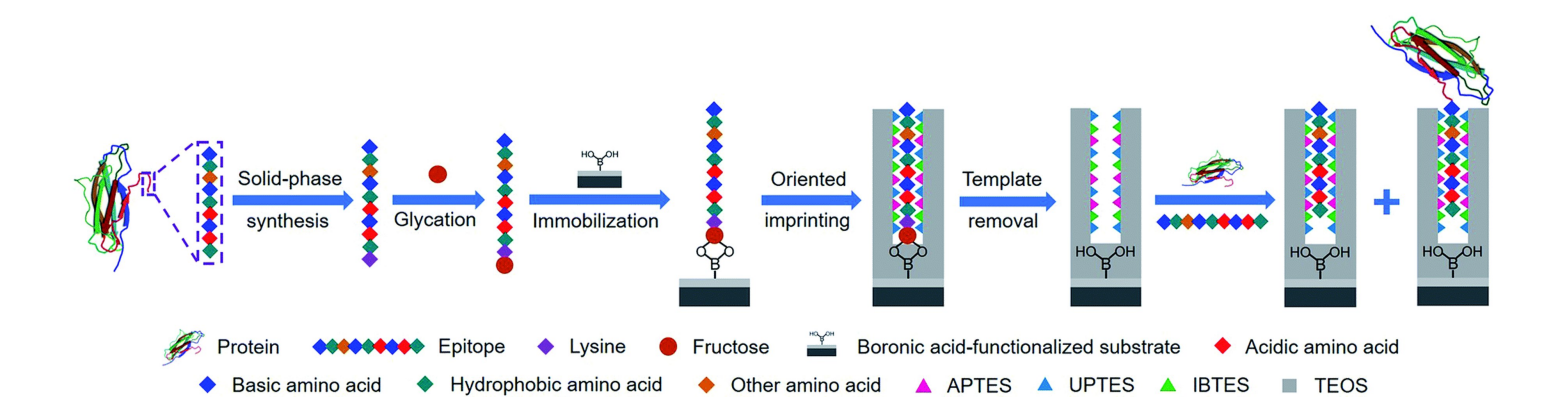
硼亲和锚定表位可控定向表面印迹技术的示意图^[33]^

在优化了功能单体比例和印迹时间后,所制备的β2微球蛋白(β-2-microglobulin, B2M)表位印迹MNPs的印迹因子(imprinting factors, IF)与*K*_d_分别达到6.5和2.08×10^-7^ mol/L;其中,IF为MIP与非印迹聚合物(non-imprinted polymers, NIP)在饱和吸附时对模板分子结合量的比值;IF值越高,则MIP的特异性越好;而*K*_d_值越低,MIP的亲和力越高。以上这些定量表征数据说明,利用硼亲和锚定表位可控定向表面印迹技术所制备的MIP具有高的特异性与亲和力。

#### 1.2.1 分子印迹及包覆策略

尽管硼亲和锚定表位可控定向表面印迹技术的便捷性与通过其所制备材料的优异性已经得到了证明,但与传统印迹方法类似,MIP的印迹空腔和非印迹表面是在相同的印迹条件下一同构建的,因此处于印迹空腔外部的非印迹表面会发生明显的非特异性吸附现象,致使在优化条件时无法同时获得最佳亲和力与特异性。为了解决上述问题,本课题组^[34]^提出了一种分子印迹及包覆策略(图4),即在定向印迹后加盖一层纳米级的化学惰性包覆层,以精准覆盖非印迹区域,这一包覆层极大抑制了由于功能单体暴露于MIP表面所导致的非特异性吸附。利用该策略所制备的包覆MIP(cMIP)可以同时获得最佳亲和力与特异性。该工作以B2M为模板制备了B2M cMIP,其*K*_d_值为1.12×10^-9^ mol/L,明显低于不含包覆层MIP的*K*_d_值(5.39×10^-9^ mol/L); cMIP的IF值为16.6,远超过了不含包覆层MIP的IF值(6.5),同时cMIP对非目标蛋白质的交叉反应性小于6.8%。以上结果说明cMIP的亲和力与特异性明显高于MIP;此外,该方法具有普适性,通过更换表位模板即可制备出针对各种目标蛋白质的cMIP。基于上述优点,分子印迹及包覆策略在复杂样品分离应用中能够发挥出极大的潜力。

**图 4:**
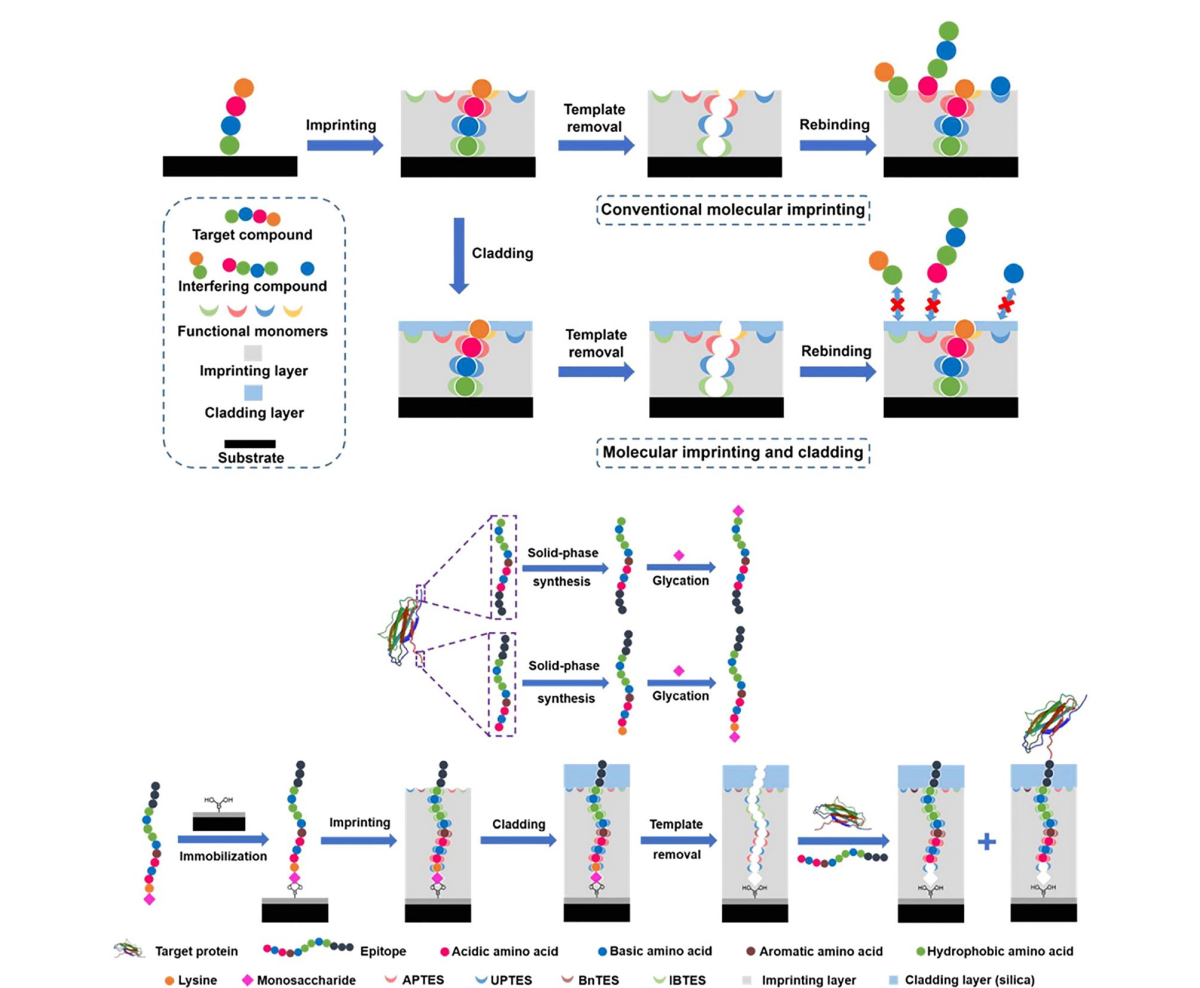
分子印迹及包覆策略的示意图^[34]^

#### 1.2.2 反相微乳液印迹

2010年Zeng等^[35]^提出了反相微乳液印迹策略。该策略先通过在亲水模板上修饰疏水基团,以确保模板分子在反相微乳液体系中能够一直保持在水相与有机相的界面上;此时,模板分子的疏水性脂肪链处于有机相中,而亲水性模板处于含有单体的水相表面,随后水相中的单体会发生聚合反应,形成对目标物具有高度特异性的MIP。反相微乳液印迹策略可以对MIP的尺寸进行精准调控,且形成的MIP具有良好的分散性,该策略已广泛应用于生物医学领域。为了将MIP与各种先进纳米材料相结合,实现更多新的应用,本课题组^[36]^提出了一种反相微乳液表位定向表面印迹及包覆方法(reverse microemulsion-confined epitope-oriented surface imprinting and cladding, ROSIC)(图5)。与先前方法不同的是,ROSIC将硅烷化试剂作为功能单体,硅烷化试剂在水解时会参与纳米颗粒的配体交换,促使纳米颗粒从油相转移至水相中以及作为内核掺入MIP中,从而赋予MIP各种功能。ROSIC具有普适性,是一种通用的纳米材料靶向识别功能化方法,其可以简单地将各种在油相中制备得到的先进纳米材料(如QDs、上转换纳米颗粒(upconversion nanoparticles, UCNPs)、超顺磁性纳米颗粒(superparamagnetic nanoparticles, SMNPs)、银纳米颗粒(silver nanoparticles, Ag NPs)等)引入至MIP中,解决了油相制备的纳米材料难以实现表面功能化的问题。此外,ROSIC还引入了前期工作中的包覆策略,极大地提高了MIP的特异性与亲和力,在生物成像及疾病诊断等领域具有良好的应用前景。

**图 5:**
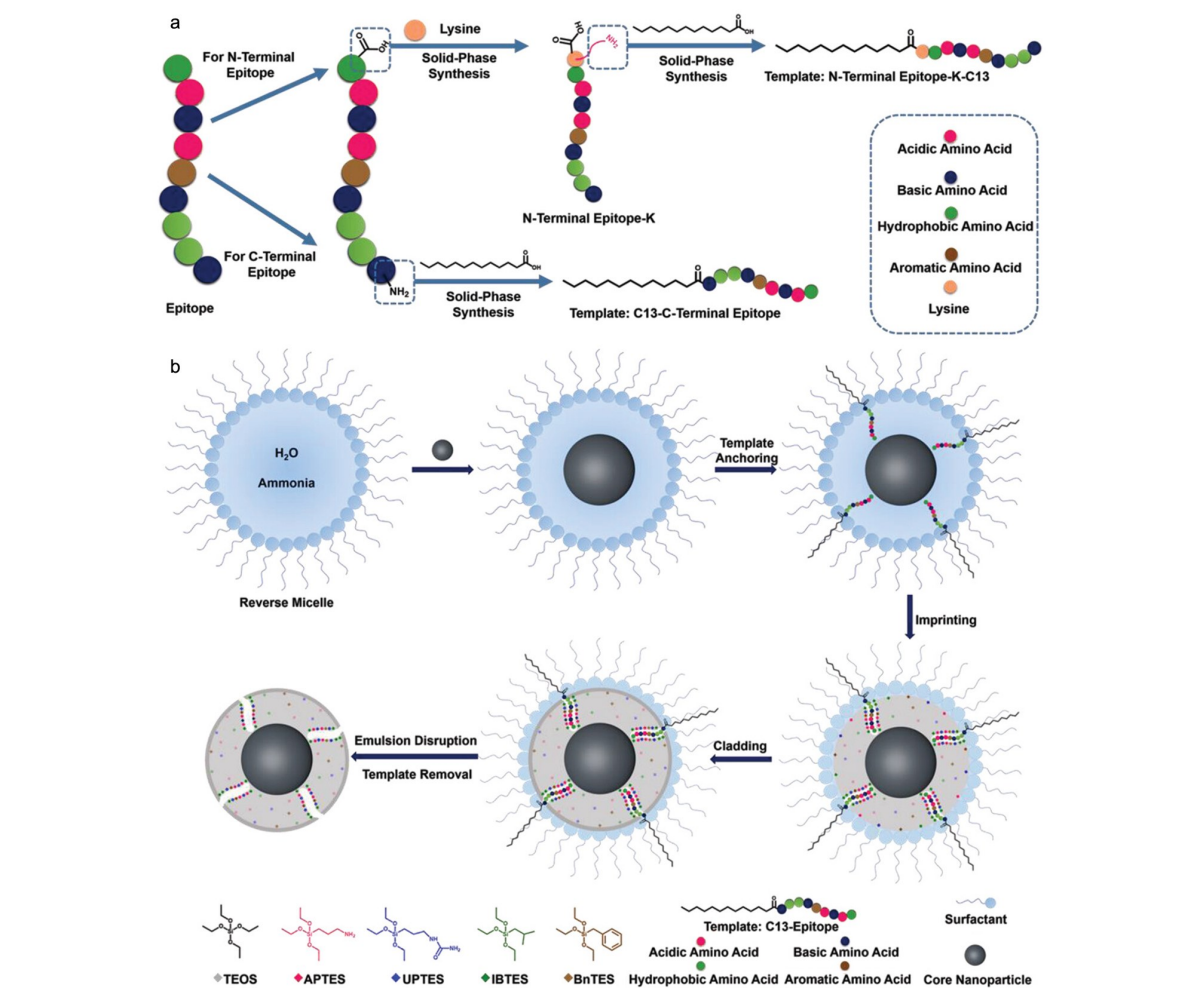
ROSIC策略的示意图^[36]^

## 2 MIT在复杂生物样品分离分析中的应用

### 2.1 色谱分离

MIP具有耐高温高压和耐酸碱的特性,与核酸和蛋白质等生物分子相比,MIP的成本更低,制备流程更加简单,物理、化学稳定性更高^[37]^。作为色谱固定相,MIP已被广泛应用于高效液相色谱(HPLC)和毛细管电色谱(CEC)中^[38,39]^。

在HPLC中,MIP一般被用于制备填充柱和整体柱。MIP填充柱一般是将MIP颗粒作为填料,通过高压气泵或高压液泵将其填充至液相钢管柱中。如Song等^[40]^通过将与多肽抗生素具有相似结构的多粘菌素E作为模板、甲基丙烯酸(methacrylic acid, MAA)和甲基丙烯酸羟乙酯(hydroxyethyl methacrylate, HEMA)作为功能单体、二乙烯基苯(divinylbenzene, DVB)和乙二醇二甲基丙烯酸酯(ethylene glycol dimethacrylate, EGDMA)作为交联剂,再经过沉淀聚合反应得到印迹微球;将该印迹微球填充至HPLC柱(100 mm×4.6 mm)中得到MIP填充柱。该MIP填充柱具有良好的稳定性和柱效,对多肽抗生素的回收率为66.7%~94.5%,相对标准偏差(RSD)低于16%,检出限(LOD)为2.0~4.0 μg/kg。与传统的预处理方法相比,该MIP填充柱能够去除样品中的杂质,并有效降低基质效应,可用于后续多肽抗生素的准确分析。MIP整体柱一般是通过在液相钢管柱中直接原位合成的。如Feng等^[41]^以MAA为功能单体、乙烯基酯树脂为交联剂、2,2-偶氮(2-甲基丙腈)(2,2'-azobis(2-methylpropionitrile), AIBN)为引发剂,通过本体聚合法制备了霉酚酸酯(mycophenolate mofetil, MMF)整体柱。该整体柱能够有效识别MMF和其代谢产物霉酚酸(mycophenolic acid, MPA),并且可在4 min内实现MMF与MPA的基线分离,分离度为2.68。此外,在以血浆样品为基质时,该整体柱对MMF的回收率达到89.17%~91.87%, RSD低于7.03%。实验结果表明,该整体柱可用于肾移植患者血浆中MMF及MPA的选择性提取与快速筛选。

CEC作为一种结合了毛细管电泳(CE)和HPLC的分析技术,具有分离效率高和选择性好等优点。基于MIP的CEC整体柱通常被用于分离手性对映体^[42]^,但近年来也有将其用在复杂生物体系分离分析领域的相关报道。如Ye等^[43]^以氧化石墨烯(graphene oxide, GO)为基质、多巴胺(dopamine, DA)为模板、MAA为功能单体制备了GO-MIP包被的毛细管;其中,GO是一种碳质纳米材料,将其作为基质能够为MIP提供高比表面积以及良好的导电率与机械强度。实验结果表明,GO-MIP包被的毛细管具有良好的稳定性,能够高效地分离、检测人血清和盐酸DA注射液中的DA及其结构类似物(如肾上腺素(epinephrine, EP)和去甲肾上腺素(norepinephrine, NE)),对目标分析物的回收率为90.3%~109.6%, RSD小于6.19%。

### 2.2 固相萃取

作为一种操作简便、回收率高、有机溶剂消耗少且易于自动化的方法,固相萃取能够从各种基质中提取分析物,已被广泛应用在样品制备中^[44,45]^。然而,传统固相萃取材料(如硅胶、活性炭与聚苯乙烯等)的选择性低,不适用于复杂样品中痕量目标物的分离富集。因此,基于MIP的固相萃取一直是分离科学的研究热点。一系列具有高选择性、高亲和力及高稳定性的新型MIP吸附材料已被用于血清、尿液和唾液等各种复杂生物样品中目标物的提取^[19,46]^,这些MIP吸附材料主要包括磁性分子印迹聚合物(magnetic molecularly imprinted polymers, MMIP)^[47,48]^、分子印迹介孔二氧化硅(molecularly imprinted mesoporous silica, MIMS)^[49⇓-51]^与分子印迹膜(molecularly imprinted membrane, MIM)^[52,53]^等。

#### 2.2.1 MMIP

基于MMIP的固相萃取方法可有效避免耗时过长的过滤和离心步骤,极大地提高了分离效率^[54,55]^。Fe_3_O_4_具有制备简单、无毒、磁性好和生物相容性高等特点,是目前使用最广泛的磁性材料,其也常被用作MMIP的磁性核心^[56]^。He等^[57]^以Fe_3_O_4_为核心(图6)、氧氟沙星(ofloxacin, OFX)为模板,通过可逆加成-断裂链转移(reversible addition-fragmentation chain transfer polymerization, RAFT)聚合反应制备出了超顺磁性的Fe_3_O_4_@MIP,并将Fe_3_O_4_@MIP直接用于人尿液中5种氟喹诺酮类药物(OFX、培氟沙星、恩诺沙星、诺氟沙星和加替沙星)的选择性富集。实验结果表明,该MIP对5种氟喹诺酮类药物的回收率为83.1%~103.1%, RSD为0.8%~8.2%。然而,由于磁性核心自身的质量较大,且无法提供任何识别位点,在一定程度上限制了MMIP的吸附能力。为了进一步提高MMIP的吸附能力,Wang等^[58]^以小粒径(5 nm)的Fe_3_O_4_为磁源,将其搭载至SiO_2_微球表面,并以DA为功能单体在SiO_2_微球表面对四环素(tetracycline, TC)进行印迹,随后对SiO_2_核心进行刻蚀,获得了芝麻球状的空心双层杂化磁性表面印迹纳米材料(HD-MMIP)。具有中空结构的HD-MMIP对TC的结合量可达70.23 mg/g,回收率为94.8%~98.5%。与上述方法不同,Yang等^[59]^利用具有丰富孔隙结构和高比表面积的石墨烯来包裹Fe_3_O_4_核心,所得到的Fe_3_O_4_@rGO@MIP具有超高的比表面积,提高了MMIP的结合性能。以DA为功能单体在皱纹花状磁性石墨烯微球的表面对牛血清白蛋白(bovine serum albumin, BSA)进行印迹,Fe_3_O_4_@rGO@MIP对BSA展现出了高度特异性,结合量可达317.58 mg/g。

**图 6:**
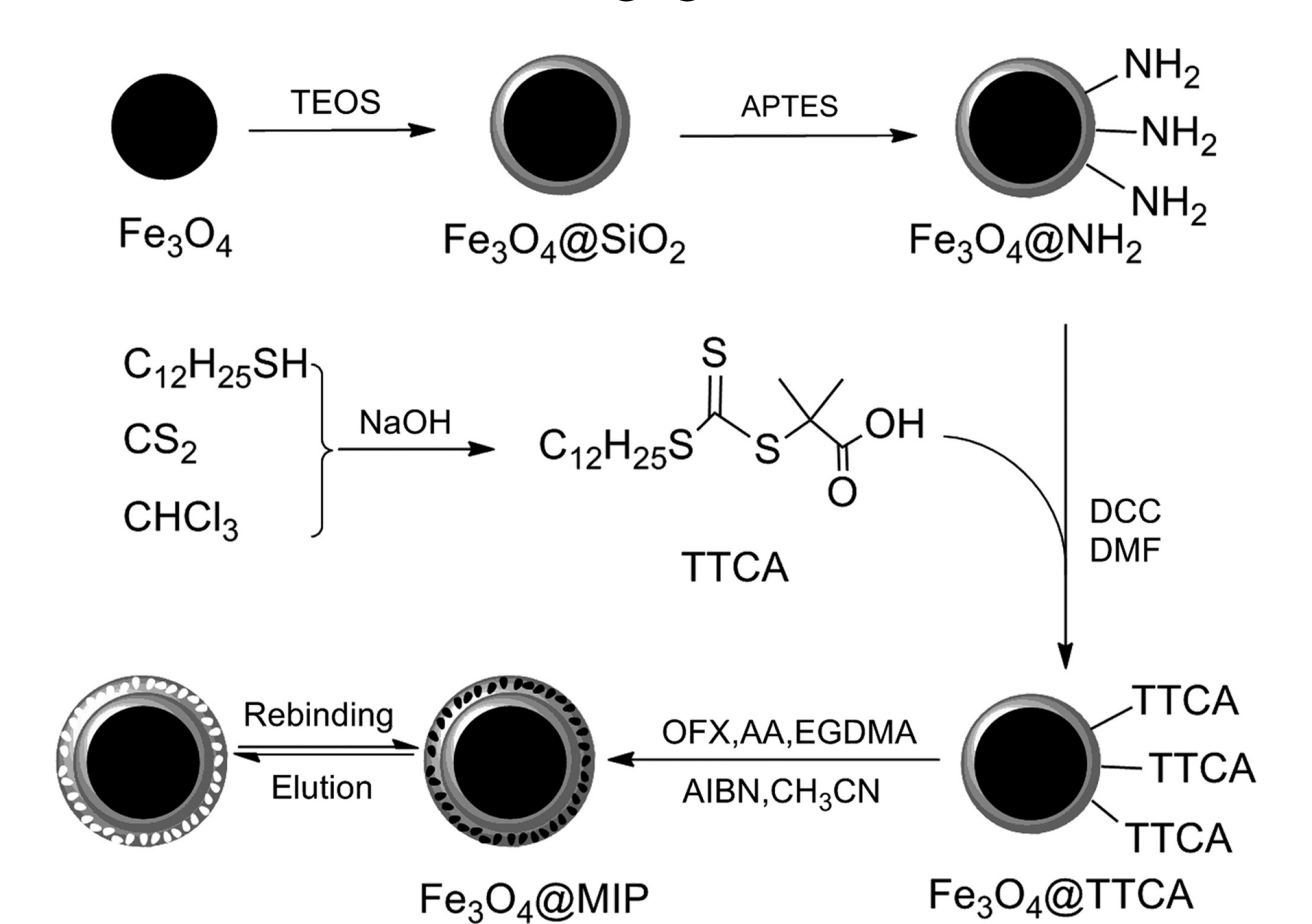
Fe_3_O_4_@MIP的制备过程^[57]^

除了单一磁响应的MMIP外,近年来还有很多研究通过在功能单体中引入刺激响应聚合物来开发出各种智能响应的MMIP^[60]^。如Wang等^[61]^以甲基丙烯酸磺基甜菜碱和对温度敏感的*N*-异丙基丙烯酰胺(*N*-isopropylacrylamide, NIPAM)为功能单体,制备了具有温度响应性的BSA-MMIP。在特定温度下(35 ℃), BSA-MMIP的印迹空腔与BSA是互补的,能够特异性地结合BSA;当BSA-MMIP处于低于临界溶液温度的环境中时,其功能单体NIPAM会产生亲水性,导致BSA-MMIP的空腔结构发生改变,不再与BSA匹配,从而有利于BSA的洗脱。Xie等^[62]^利用具有光响应功能的4-[4-(丙烯酰氧基)苯基偶氮]苯甲酸(4-[(4-prop-2-enoyloxyphenyl)diazenyl]benzoic acid, MAPASA)制备了用于特异性识别牛血红蛋白(bovine hemoglobins, BHb)的MMIP,其中MAPASA中的偶氮苯发色团在光诱导下会发生顺反异构化,从而导致印迹位点产生变化;当处于440 nm波长光照射下时,MMIP对BHb具有良好的结合能力,而在365 nm波长光照射下,MMIP中的印迹位点发生变化,导致其对BHb的结合能力减弱,致使目标物从印迹位点上脱落。实验结果表明,该MMIP具有优异的磁分离以及光响应性能,能够用于牛血清中BHb的特异性提取。

#### 2.2.2 MIMS

MIMS具有超高的比表面积和孔隙率,可以有效提高分子靶标的结合能力及位点可及性^[63]^。MIMS常被用于分离富集各种复杂样品中不同类型的分析物,如天然化合物、多肽和蛋白质等^[64]^。Susanti等^[65]^以硫酸沙丁胺醇为模板、十六烷基三甲基溴化铵(cetyltrimethylammonium bromide, CTAB)为表面活性剂、TEOS和甲基三乙氧基硅烷(methyltriethoxysilane, MTES)为功能单体,制备了可用于提取血清中沙丁胺醇的MIPMS。该MIPMS对沙丁胺醇的结合量为93.4 μg/g,回收率为104.79%。Yan等^[26]^同样以SiO_2_颗粒为基质、转铁蛋白(transferrin, Trf)为模板制备了含有室温磷光(room-temperature phosphorescence, RTP)量子点(PQDs)的SiO_2_-PQDs-MIPs(图7)。SiO_2_-PQDs-MIPs对Trf的LOD、RSD、IF和回收率分别为0.014 μmol/L、3.23%、3.01和94.5%~102%,实验结果表明,SiO_2_-PQDs-MIPs能够从尿液和血清样本中特异性提取Trf。

**图 7:**
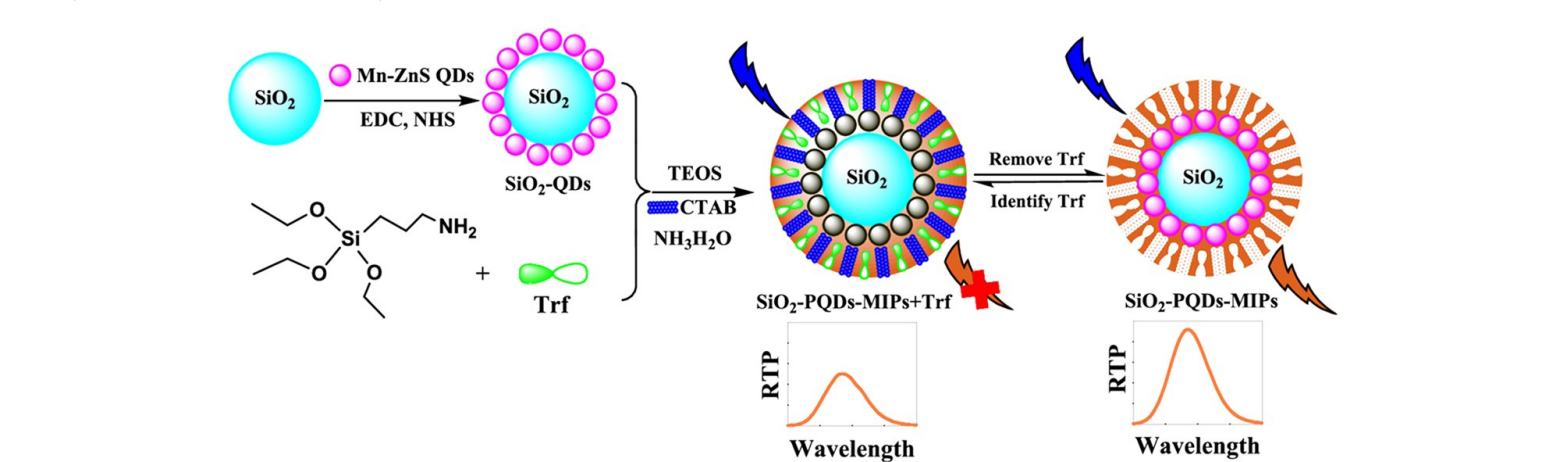
SiO_2_-PQDs-MIPs的制备流程及Trf识别原理^[26]^

为进一步提高MIMS的识别性能和制备方法的普适性,本课题组^[66]^提出了一种双模板对接定向分子印迹(dual-template docking oriented molecular imprinting, DTD-OMI)策略,该策略利用静电相互作用将带有负电荷的单磷酸腺苷(adenosine monophosphate, AMP)模板分子锚定在带有正电荷的棒状CTAB胶束上,并形成双模板复合物,随后以3-缩水甘油氧基丙基三乙氧基硅烷((3-glycidyloxypropyl)triethoxysilane, APBA-GPTES)为功能单体、TEOS为交联剂直接在双模板复合物四周进行聚合反应,得到AMP印迹的美孚晶体材料-41(mobil crystalline materials-41, MCM-41)介孔SiO_2_纳米颗粒(adenosine monophosphate mesoporous silica nanoparticles, AMP-MSN)。在进行聚合反应时,AMP模板分子会定向锚定在CTAB表面,因此所有的印迹空腔都整齐地分布在AMP-MSN的介孔壁上,从而提高了材料的识别与结合量。该AMP-MSN的IF值可达43.6,交叉反应性低于23%,结合量为191.7 μmol/g,能够有效地从人尿液中特异性富集AMP。同时,该策略还具有普适性,可广泛应用于各种带电目标分子的印迹,而对于不带电的目标分子也可以使用带电类似物作为虚拟模板来进行印迹。除此之外,本课题组^[67,68]^还基于该策略,制备了用于富集磷酸化肽以及阿马道里(Amadori)化合物的分子印迹介孔SiO_2_纳米颗粒(molecularly imprinted mesoporous silica nanoparticles, MIMSNs)。

#### 2.2.3 MIM

MIM最早出现于1990年^[69]^, MIM结合了膜分离技术与MIT的优点,如特异性高、易于连续化及使用简便等^[70]^。MIM的印迹位点均匀地分布在膜表面,与一般的MIP相比,MIM具有更高的结合能力和传质效率,已被广泛应用于复杂生物样品中的蛋白质分离。但在MIM的制备过程中,蛋白质模板的体积大且结构复杂,常常会与交联剂紧密结合,导致模板难以去除。为了解决该问题,Armutcu等^[71]^将BHb吸附在ZnO颗粒上,以乙二醇二甲基丙烯酸酯为功能单体和交联剂,通过本体聚合制备了聚丙烯酸MIM,随后去除BHb-ZnO颗粒,在本体聚合物膜上留下有效的印迹空腔,最终得到BHb-MIM。在印迹过程中,ZnO颗粒不仅可以保留聚合物网络中的印迹空腔,还有利于模板的去除及目标物的再结合。所制备的BHb-MIM对BHb具有高特异性,分离因子(separation factor, SF)为6.78,吸附容量(*Q*_max_)为73.53 mg/g。此外,如何在印迹过程中确保模板蛋白质结构的完整性也是MIM制备过程中的关键问题。为此,Fan等^[72]^以离子液体1-乙烯基3-(2-氨基-2-氧代乙基)氯化咪唑(1-vinyl-3-aminoformylmethyl imidazolium chloride, [VAFMIM]Cl)为功能单体、素蛋白和海藻酸钠为机械改性剂、*N*,*N*-亚甲基双丙烯酰胺(methylene-bis-acrylamide *N*,*N*'-methylenebis(2-propenamide), MBA)为交联剂、过硫酸铵(ammonium persulphate, APS)为引发剂,制备了能够用于特异性识别BHb的水凝胶MIM;其中,离子液体[VAFMIM]Cl能够与蛋白质形成多重相互作用,有助于维持蛋白质的结构与活性;机械改性剂的加入使水凝胶MIM具有超高的机械强度,解决了传统水凝胶结构脆弱的问题。此外,该水凝胶MIM具有超大的孔隙结构以及良好的传质效率,能够有效地从牛血清中特异性提取BHb,结合量为121.8 mg/g, SF为7.34。

### 2.3 疾病诊断

体液中的异常疾病标志物是疾病诊断的重要工具,然而痕量的疾病标志物和复杂的样品基质使得临床疾病诊断变得困难,因此亟需一种高特异性与高灵敏度的检测方法来解决该问题。具有高特异性与高亲和力的MIP可以极大降低血液、尿液、泪液或汗液等复杂生物样品的基质效应,并能够特异性地提取和富集疾病标志物,以便用于后续检测。此外,与天然识别元件(如酶、抗原、抗体、DNA或其他受体)相比,MIP具有更优异的稳定性和更低廉的成本^[73]^。因此,近年来基于MIP的各类疾病标志物检测技术,特别是糖蛋白检测技术受到了广泛关注。Balayan等^[74]^开发出了一种基于MIT和电化学平台的肿瘤坏死因子α(tumor necrosis factor-α, TNF-α)检测方法。以TNF-α为模板、MAA为功能单体,制备了TNF-α MIP生物传感器,并将其与MoS_2_纳米片及Fe_3_O_4_@SiO_2_纳米颗粒一同修饰到工作电极上;其中,TNF-α MIP提高了传感器的特异性,而二维MoS_2_纳米片和Fe_3_O_4_@SiO_2_纳米颗粒则增加了工作电极的电导率。该TNF-α MIP生物传感器具有电压要求低、样品量小、灵敏度高等优势,对TNF-α的检测范围为0.01 pmol/L~100 nmol/L,并能够在3 min内实现TNF-α的快速检测。Siavash等^[75]^开发了一种纳米结构微流控电化学多路复用装置(NFluidEX),用于唾液和全血中病毒蛋白及特异性抗体的定量检测。该装置主要是通过电聚合将邻苯二胺(*o*-PD)固定在电极表面,再分别以新型冠状病毒-2(SARS-CoV-2)蛋白、免疫球蛋白G-受体结合区域(immunoglobulin G-receptor-binding domain, IgG-RBD)和免疫球蛋白M-受体结合区域(immunoglobulin M-receptor-binding domain, IgM-RBD)为模板制备了印迹聚合物薄膜。当电极外的印迹空腔与病毒或抗体结合时,NFluidEX能够检测到相应的阻抗幅度变化,从而实现唾液中病毒以及血液中IgG和IgM抗体的平行定量。NFluidEX能够有效区分COVID的阳性和阴性,其灵敏度与特异性均达到了100%。虽然电化学传感器与MIT的结合是一种简单、快速、便携的疾病标志物检测方法,但要将其应用到临床诊断上仍然会面临巨大的挑战,包括检测的重现性以及MIP电子传递效率相对较差所导致的电导率低等问题^[76,77]^。

除分子印迹电化学传感器外,本课题组^[78]^发展了一种基于分子印迹与表面拉曼增强(surface-enhanced raman scattering, SERS)的检测方法,称为双重MIP等离子体免疫夹心测定法(dual MIP-based plasmonic immunosandwich assay, duMIP-PISA)。以癌症标志物神经元特异性烯醇化酶(neuron-specific enolase, NSE)为例,duMIP-PISA依赖于两种不同的MIP识别,一种用于NSE的捕获,另一种则用于标记。通过硼亲和锚定表位可控定向表面印迹方法,以NSE的C端与N端表位肽为模板,分别制备了用于捕获NSE C端表位肽的印迹金纳米颗粒(Au NPs)基板以及用于识别NSE N端表位肽的标记粒子(Ag/PATP@SiO_2_ NPs)。得益于对不同靶点的双重识别,所制备出的两种MIP对NSE均展现出了极高的特异性,交叉反应性低于6.7%, *K*_d_值为3.4×10^-9^mol/L。此外,基板、被捕获蛋白质与标记粒子间形成了三明治夹心结构,在激光照射下,Au NPs与Ag NPs会产生等离激元增强拉曼散射(plasmon-enhanced raman spectroscopy, PERS)效应,使得拉曼信号增强多个数量级。该方法成功实现了血清中NSE的检测,且与商业化的ELISA检测试剂盒相比,duMIP-PISA具有更简单的流程、更低的样品体积要求与更宽的检测范围。除了糖蛋白的表达水平外,其糖基化的异常也与疾病发生有着密切的相关性,因此对糖蛋白标志物的糖基化表达情况进行检测,能够为疾病的精准诊断提供重要依据。为此,本课题组^[79]^在duMIP-PISA的基础上提出了一种三重MIP等离子体免疫夹心测定法(triMIP-PISA),该方法能够对所捕获的糖蛋白标志物及其末端单糖进行同时标记。以甲胎蛋白(alphafetoprotein, AFP)为蛋白标志物,制备了可同时标记AFP和甲胎蛋白异质体(AFP-L3)的表位MIP,以及只能标记AFP-L3末端核心岩藻糖(fucose, Fuc)的Fuc-MIP,实现了血清中AFP和AFP-L3的同时、快速PERS检测,并能够有效地区分肝细胞癌(HCC)患者与健康对照者。此外,与小扁豆凝集素(LCA)相比,Fuc-MIP对Fuc具有更高的特异性与亲和力。HCC患者体内AFP糖基化的异常表达除了Fuc外还有末端唾液酸(sialic acid, SA),为此本课题组^[80]^进一步开发了基于MIP的双模态比例免疫测定法(图8)。

**图 8:**
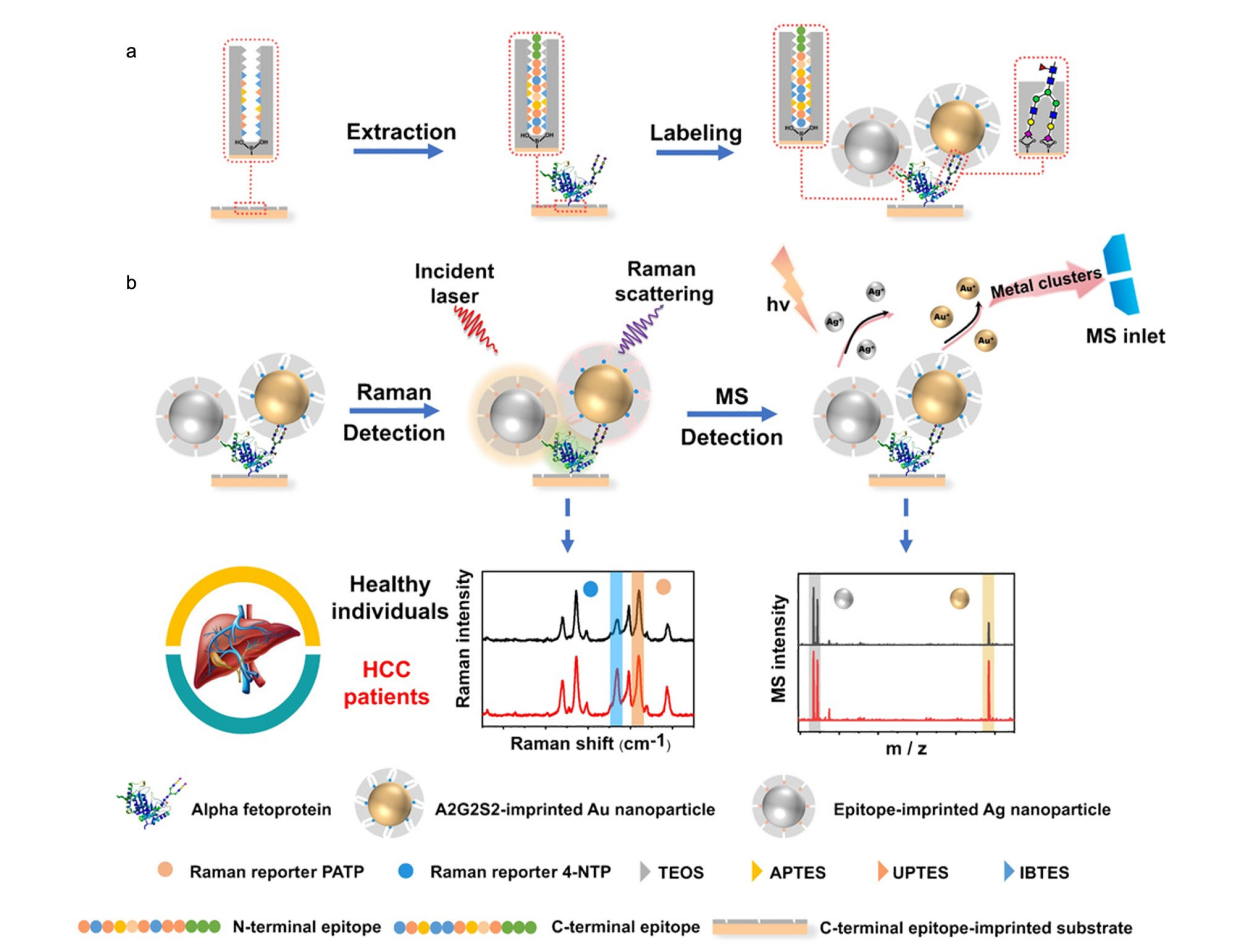
双模态比例免疫测定法的示意图^[80]^

该方法将Trf上的A2G2S2糖链以及AFP的N端作为模板分子,分别制备了能够有效识别AFP上含有SA和/或Fuc聚糖子集(Gm)的标记粒子(A2G2S2-imprinted Au NP),以及AFP N端的标记粒子(epitope-imprinted Ag NP),并结合了SERS与激光解吸电离质谱(laser desorption ionization-mass spectrometry, LDI-MS)检测技术。该方法中的SERS检测部分与triMIP-PISA方法类似,通过形成三明治夹心结构产生PERS效应以检测拉曼信号,从而获得AFP上Gm与AFP(AFP-Gm/AFP)的信号比值;在LDI-MS检测方面,通过激光照射夹心结构时产生的银纳米团簇(*m/z* 107)和金纳米团簇(*m/z* 197)质谱信号,可获得第二组AFP-Gm/AFP的质谱信号比值。该双模态比例免疫测定法可以提供两个独立测量的AFP-Gm/AFP比值,允许相互比对,并能够提供可靠的结果。与ELISA相比,该方法对HCC患者的诊断更加准确,区分度更高,在疾病诊断等许多重要应用中有着广阔的应用前景。

### 2.4 生物成像

MIP具有靶向各种目标分子的能力,且易于引入各种纳米材料(QDs、UCNPs等)或染料分子^[81⇓⇓-84]^。因此,近年来各类功能化的MIP在生物成像领域受到了关注^[85]^。将MIP应用在生物成像领域的一系列研究最早出现在2015年^[86]^。Kunath等^[87]^通过沉淀聚合反应成功制备了葡萄糖醛酸(glucuronic acid, GlcA)印迹的荧光MIP,并将其应用于人角质形成细胞和人体皮肤组织的生物成像。该MIP中所掺杂的罗丹明B染料可用于荧光成像,而GlcA印迹空腔则用于特异性靶向透明质酸。结果表明,该荧光MIP能够清晰地展示透明质酸的定位与分布。随后Panagiotopoulou等^[88]^进一步在对应的MIP中分别引入具有红色与绿色荧光的量子点,制备了MIP-QDs,并成功将其用于人角质形成细胞上GlcA和*N*-乙酰神经氨酸(NANA)的双重成像(图9)。

**图 9:**
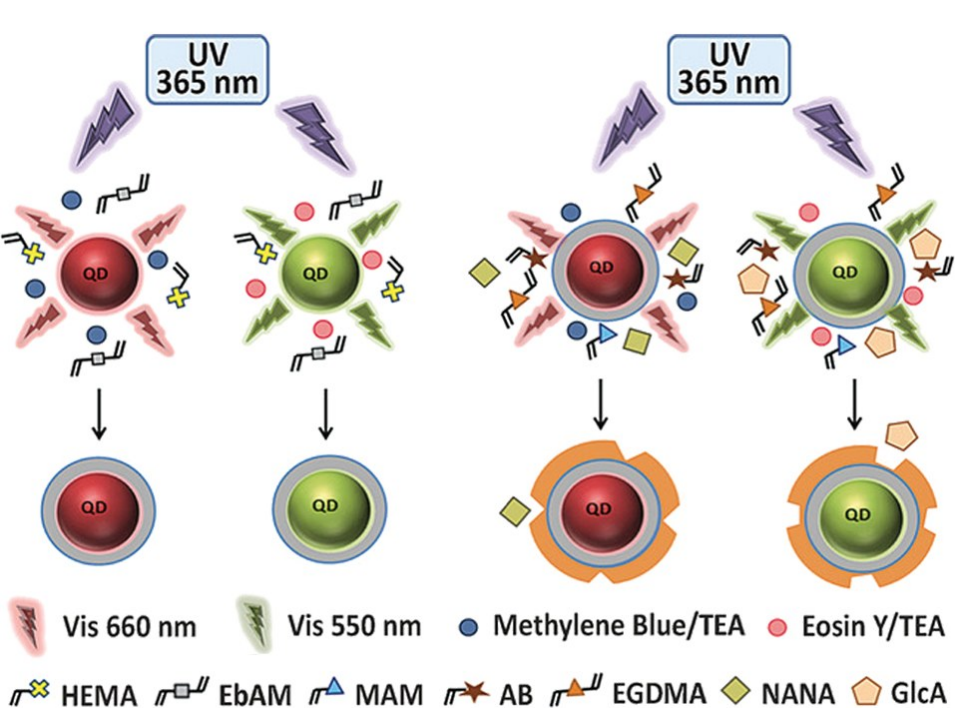
MIP-QDs的合成路线^[88]^

本课题组^[89]^制备了掺有3种不同荧光发射波长量子点的单糖印迹MIP,并实现了癌细胞中SA、Fuc与甘露糖(mannose, Man)的多重成像。该技术可以提供癌细胞中3种目标单糖表达水平的相关信息,能够用于揭示不同细胞系之间的异同,并识别特定的癌细胞,有望成为区分癌细胞系的有效手段。

除了基于荧光MIP的生物成像技术外,本课题组^[90]^还提出了一种基于MIT和SERS的拉曼成像技术。利用含有拉曼报告分子和Ag NPs核心的SA印迹MIP,实现了对肝癌细胞与肝癌组织的高特异性SERS成像。此外,本课题组^[91]^通过反相微乳液印迹策略制备了能够特异性识别酪氨酸磷酸化的SERS标签MIP,并将其成功用于肝癌细胞和肝癌组织中磷酸酪氨酸的SERS成像。与荧光成像技术相比,基于SERS的拉曼成像技术具有更高的灵敏度和光稳定性以及更优异的多重检测能力。将MIT与SERS成像技术相结合,有望为癌症诊断及相关研究开辟一条新的途径。

Cecchini等^[92]^以血管内皮生长因子(hVEGF)的C端九肽为模板,通过固相合成制备了QDs偶联的MIP(anti-hVEGF MIP)。在斑马鱼胚胎中,anti-hVEGF MIP可实现对过表达hVEGF肿瘤团块以及异种移植人类黑色素瘤细胞的特异性靶向成像。尽管MIP已在体内成像应用中展现出了极大的潜力,但要将其应用在临床上还需进一步提高成像信号及生物相容性。

### 2.5 蛋白质组学研究

蛋白质组学的本质是对细胞和组织中存在的蛋白质进行大规模的分析鉴定,包括表达水平、翻译后修饰及相互作用等,是一门可用于细胞代谢与疾病过程的新兴研究^[93]^。质谱是蛋白质组学研究的核心技术,但其存在一些缺点,包括对生物样品中复杂基质干扰的耐受性差,以及对低丰度蛋白质和肽的灵敏度低等。在进行质谱分析前,样品的特异性富集与高效净化尤为重要^[12]^。因此,具有高特异性与高亲和力的MIP在蛋白质组学研究中具有极大的应用潜力。在靶向蛋白质组学分析中,MIP一般用于肽或完整蛋白质的富集^[93]^。Yang等^[94]^将乙酰化赖氨酸和丙氨酸残基的二肽(KacA)作为模板,制备了一种能够特异性捕获赖氨酸-乙酰化肽(Kac-peptides)的Mono-MIP,实现了细胞裂解液中Kac肽的富集。纳米液相色谱-串联质谱(nano LC-MS/MS)分析结果表明,经Mono-MIP富集后,在细胞裂解液中鉴定出的Kac肽、位点和蛋白质的数量明显增加,并且部分Kac肽只有通过Mono-MIP富集后才能被鉴定到。Lü等^[95]^通过DTD-OMI与溶胶-凝胶工艺,以Fe_3_O_4_为核心制备了焦磷酸印迹磁性亲和微球,实现了经胰蛋白酶消化后的脱脂牛奶中及人血清样品中磷酸肽的快速、有效捕获。利用上述MIP对脱脂牛奶进行富集后,经质谱检测出的磷酸肽由1种增加至9种,并且人血清中磷酸肽的质谱信号强度也明显增加。对于完整蛋白质,Wan等^[96]^以Lys为模板,将其固定在核壳磁性纳米颗粒上,再通过与DA共聚得到了能够特异性磁富集Lys的MIP。该MIP对Lys具有高亲和力与高结合力,最大吸附量和Langmuir常数分别为202.02 mg/g和29.61 mL/mg。在对蛋清样品进行富集后,利用基质辅助激光解吸电离-飞行时间质谱(MALDI-TOF MS)进行检测,Lys的质谱信号有明显增强。MIP除了能够对完整蛋白质进行特异性富集外,还能用于高丰度蛋白质的去除,从而提高质谱对低丰度蛋白质的检测灵敏度^[97⇓-99]^。Yang等^[100]^以人血清为印迹模板,制备了人血清MIP冷冻凝胶,用于去除血清中的高丰度蛋白质。值得注意的是,在制备人血清MIP冷冻凝胶时,并非血清中的所有蛋白质都会被印迹,只有高丰度蛋白质能够起到模板的作用。使用nano LC-MS/MS对原始血清与经过MIP耗竭的血清进行检测,通过比较分析发现,耗竭血清中白蛋白、免疫球蛋白、血红素结合蛋白等高丰度蛋白质的信号强度均降低了10倍以上,而其他丰度较低蛋白质(如补体C1q、载脂蛋白A与脂蛋白A等)的信号强度明显增加。

此外,MIP还能用于外泌体的高效捕获,以进行蛋白质组学分析。Liu等^[101]^利用本体印迹策略,将与外泌体具有相同尺寸且均有带负电荷的SiO_2_作为虚拟模板,制备了能够在尿液和培养基中捕获外泌体的MIP。经质谱检测发现,利用该MIP在尿液中富集到的Top100外泌体蛋白质丰度由1.85%增加至9.66%,而在细胞培养基中富集到的Top100蛋白质丰度为28.4%。此外,该MIP在捕获外泌体方面具有与商业试剂盒相当的效果,有望用于复杂样品中外泌体的蛋白质组学研究。综上,表1汇总了MIT在复杂生物样品分离分析中的应用,包括制备方法、模板分子、样品类型以及分离分析模式等。

**表 1:** MIT在复杂生物样品分离分析中的应用

Application	Materials	Method	Templates	Samples	Separation and detection modes	Ref.
Chromatog- raphy	MIP filled column	precipitation poly- merization	polymyxin E	muscle samples including beef, pork and chicken	LC-MS/MS	[40]
	MIP monolithic column	bulk polymerization	MMF	plasma	HPLC	[41]
	GO-MIP	bulk polymerization	DA	human serum and DA hydrochloride injection	CEC	[43]
SPE	OFX-imprinted Fe_3_O_4_@MIP	RAFT polymerization	OFX	human urine	magnetic separation	[57]
	HD-MMIP	surface imprinting	TC	milk sample	magnetic separation	[58]
	Fe_3_O_4_@rGO@MIP	surface imprinting	BSA	fetal bovine serum	magnetic separation	[59]
	BSA-MMIP	surface imprinting	BSA	protein solutions	magnetic separation	[61]
	BHb-MMIP	surface imprinting	BHb	bovine serum	magnetic separation	[62]
	MIPMS	direct incorporation method	salbutamol sulfate	serum	centrifuge	[65]
	SiO_2_-PQDs-MIPs	surface imprinting	Trf	urine and serum	fluorescence spectro- photometer	[26]
	AMP-imprinted MSN	DTD-OMI	AMP	urine	micellar electrokinetic chromatography	[66]
	BHb-MiM	surface imprinting	BHb	protein solutions	membrane separation	[71]
	MIM	bulk polymerization	BHb	bovine serum	membrane separation	[72]
Disease diagnosis	TNF-α MIP	bulk polymerization	TNF-α	-	electrochemical biosensor	[74]
	NFluidEx	electro-polymeriza- tion	IgG/IgM	saliva and blood	electrochemical biosensor	[75]
	NSE-imprinted Ag/PATP@SiO_2_ NPs and NSE-imprinted AuNP SAM-coated glass substrates	surface imprinting	NSE epitope	human serum	Raman spectrometer	[78]
	AFP/Fuc-imprinted labeling SERS tag and AFP-imprinted substrate	surface imprinting	AFP epitope and Fuc	human serum	Raman spectrometer	[79]
	AFP/A2G2S2-imprinted Au/Ag NP and AFP-imprinted substrate	surface imprinting	AFP epitope and A2G2S2	human serum	Raman spectrometer and LDI-MS	[80]
Bioimaging	GlcA-imprinted MIP	precipitation polymerization	GlcA	keratinocytes and human skin specimen	confocal microscopy	[87]
	MIPNANA-QDs MIPGlcA-QD	photopolymerization	GlcA and NANA	keratinocytes	confocal microscopy	[88]
	SA/Fuc/Man-imprinted QD	surface imprinting	SA, Fuc and Man	cells	confocal microscopy	[89]
	SA-imprinted SERS nanotags	surface imprinting	SA	liver cancer cells and tissues	Raman spectroscopy	[90]
	Anti-pY-cMIP SERS nanotags	surface imprinting	phosphotyrosine	liver cancer cells and tissues	Raman spectroscopy	[91]
	anti-hVEGF-MIP	surface imprinting	hVEGF epitope	zebrafish embryos	confocal microscopy	[92]
Proteomics	Mono-MIP	bulk polymerization	KacA	cell lysates	nano-LC-MS/MS	[94]
	pyrophosphate-imprinted MMSMs	DTD-OMI	pyrophosphate	digested nonfat milk and human serum	MALDI-TOF MS	[95]
	Lys-MIP	surface imprinting	Lys	chicken egg white	MALDI-TOF MS	[96]
	cryogel MIP	bulk polymerization	human serum	human serum	nano-LC-MS/MS	[100]
	MIP	dull template method	silica nanoparti- cles	urine and HeLa-CCM	LC-MS	[101]

MMF: mycophenolate mofetil; GO: graphene oxide; DA: dopamine; CEC: capillary electrochromatography; OFX: ofloxacin; RAFT: reversible addition-fragmentation chain transfer; HD: hollow double; MMIP: magnetic molecularly imprinted polymers; TC: tetracycline; rGO: reduced graphene oxide; BSA: bovine serum albumin; BHb: bovine hemoglobins; MIPMS: molecularly imprinted mesoporous silica; PQDs: phosphorescence quantum dots; Trf: transferrin; AMP: adenosine monophosphate; MSNs: mesoporous silica nanoparticles; DTD-OMI: dual-template docking oriented molecular imprinting; MIM: molecularly imprinted membrane; TNF-α: tumor necrosis factor-α; IgG: immunoglobulin G; IgM: immunoglobulin M; NSE: neuron-specific enolase; PATP: *p*-aminothiophenol; AFP: alphafetoprotein; LDI: laser desorption ionization; Fuc: fucose; GlcA: glucuronic acid; NANA: *N*-acetylneuraminic acid; SA: sialic acid; Man: mannose; SERS: surface-enhanced raman scattering; Anti-pY-cMIP: phosphotyrosine-specific cladding molecularly imprinted polymer; hVEGF: human vascular endothlial growth factor; KacA: acetylated lysine dipeptide; MMSMs: magnetic mesoporous silica microspheres; Lys: lysozyme; CCM: cell culture medium; -: not given.

## 3 结论与展望

本综述对新型印迹策略以及MIT在复杂生物样品分离分析中的相关应用进行了概述。MIP具有特异性高、亲和力强、稳定性好、制备简单以及成本低廉等优势,已被广泛应用于色谱分离和固相萃取中。此外,随着各种新型印迹技术的开发,将MIP与一系列先进纳米材料相结合,并利用高灵敏检测技术,可在疾病诊断、生物成像和蛋白质组学等生物医学领域中实现多种应用。然而,MIP在上述领域中的应用研究还处于早期阶段,仍存在很多挑战^[18]^。首先,在疾病诊断中,基于MIP诊断平台的选择性还有待进一步提高,以满足复杂生物样品中痕量标志物的精准诊断需求,特别是对于糖类等小分子目标分析物^[73]^。糖蛋白的糖基化异常与癌症等病理状况密切相关,糖蛋白的糖基化分析对疾病诊断具有重要意义^[102⇓-104]^。单糖间的结构差异并不明显,目前MIP对单糖的识别性能虽已超越凝集素,但要实现对糖型的精确识别还需进一步提高其选择性。其次,引入了发光纳米材料或报告分子的MIP在体外生物成像领域的应用已被广泛报道,但用于体内成像的研究还相对较少,这是因为将MIP应用于体内成像时,需同时满足可生物降解、低生物毒性以及强成像信号等条件^[85]^,而目前用于生物成像的MIP尚未达到这些要求。将MIP应用于复杂生物样品的蛋白质组学研究时,同样还存在着特异性不足的问题^[12]^,特别是低丰度蛋白质的共耗竭问题,即在去除高丰度蛋白质的同时会不可避免地导致低丰度蛋白质的损失^[105]^。此外,随着疾病诊断、生物成像与蛋白质组学等领域的发展,用于外泌体、细菌或细胞等超大模板分离识别的MIP备受关注。尽管针对小分子、多肽和蛋白质的印迹方法已经相对成熟,但由于细胞等超大模板分子具有复杂性、流动性以及巨大的尺寸,对其进行印迹仍然具有挑战性^[106]^。针对该类模板表面的复杂成分来设计MIP对于最终的目标识别至关重要,而目前只发现了少数膜蛋白和聚糖可作为识别靶点用于上述模板的识别;并且,该类模板的巨大尺寸与流动性会导致难以产生选择性互补的识别位点^[107]^,因此有待发展新型的印迹策略以形成高选择性与形状大小完整的印迹空腔。最后,尽管MIP在复杂生物样品分离中的应用已经相对成熟,特别是在高通量分析中优势明显,但仍存在制备条件精细、产量较低以及制备技术不够成熟等问题^[108]^,且目前大多处于实验室阶段,距离商业化大规模应用还有很大距离。

综上所述,未来MIT的发展会集中在以下几个方面:(1)发展新型印迹策略,以进一步提高选择性与亲和力,降低非特异性吸附,从而实现目标物的精准识别;(2)发展高生物相容性或可生物降解的新型功能单体以及引入先进纳米材料,以实现MIP的体内成像等应用;(3)在MIP的制备技术中引入基于理论模拟的条件优选以及自动化合成等策略,以实现规模化生产,同时进一步促进MIP的产业化发展。
